# Light Exposure as a Modifiable Determinant of Mental Health

**DOI:** 10.1007/s11920-026-01671-7

**Published:** 2026-04-28

**Authors:** Manuel Spitschan, Johannes Zauner

**Affiliations:** 1 Department Health and Sports Sciences, TUM School of Medicine and Health, Chronobiology & Health, Technical University of Munich, Munich, Germany; 2 Max Planck Institute for Biological Cybernetics, Max Planck Research Group Translational Sensory & Circadian Neuroscience, Tübingen, Germany; 3 TUM Institute for Advanced Study (TUM-IAS), Technical University of Munich, Garching, Germany; 4 TUMCREATE Ltd., Singapore, Singapore

**Keywords:** Light Exposure, Mental Health, Circadian Rhythms, Exposome, Non-Visual Photoreception, Light-Based Interventions

## Abstract

**Purpose of Review:**

Light is a fundamental environmental signal that shapes human physiology, behaviour, and mental health. Beyond vision, light exposure regulates circadian rhythms, sleep, neuroendocrine function, arousal, and brain circuits implicated in emotional regulation. This review synthesizes recent evidence linking light exposure to mental health and argues that light should be conceptualized as a core, modifiable component of the mental health exposome – the cumulative, dynamic set of environmental influences shaping mental health across the lifespan.

**Recent Findings:**

Recent randomized controlled trials using light therapy, large-scale epidemiological studies, and neurophysiological investigations demonstrate that habitual patterns of daytime and nighttime light exposure are associated with a broad range of mental health outcomes. Higher daytime light exposure is generally associated with better mood and lower depressive symptomatology, whereas greater exposure to light at night is linked to increased risk of depression, anxiety, and sleep disturbance. Advances in wearable light measurement and digital phenotyping now enable precise characterization of individual light environments, supporting observational studies and improving dose verification in light-based interventions. Emerging neurobiological evidence further suggests that light can influence affective brain circuits through pathways that extend beyond sleep and circadian regulation.

**Summary:**

Together, converging evidence positions light exposure as a biologically potent and highly modifiable determinant of mental health operating across multiple temporal scales, from acute alerting effects to longer-term circadian and behavioural adaptation. Conceptual challenges remain, including bidirectionality between light exposure and mental health and limitations in causal inference. Nevertheless, improved measurement technologies and personalized, just-in-time intervention strategies open new opportunities for integrating light exposure into psychiatric research, prevention, and clinical practice.

## Introduction

Mental health disorders are thought to emerge from the dynamic interaction between biological vulnerability and environmental exposure. While psychiatry has traditionally focused on genetic risk, neurochemistry and psychosocial stressors, there is growing recognition that everyday environmental factors play a substantial role in shaping mental health trajectories. Among these factors, light exposure occupies a unique and increasingly prominent position [1–3].

Light is a pervasive environmental signal with profound biological effects extending well beyond vision [1, 3–6]. Exposure to light in the evening and at night can reduce or fully suppress the production of the endogenous hormone melatonin, and additionally, lead to delays of the circadian clock [7]. Light exposure during the day can boost alertness [8, 9], and also offset the negative effects of light on melatonin production and circadian clock. Through this “non-visual” influence on circadian rhythms, sleep–wake regulation, hormonal secretion, arousal and brain function, light directly interfaces with processes that are centrally implicated in mental health.

At the same time, modern lifestyles have fundamentally altered human light exposure patterns, characterized by reduced exposure to bright natural light during the day and increased exposure to artificial light at night [6, 10]. These changes have occurred rapidly on an evolutionary timescale, raising concerns about their consequences for mental wellbeing.

In parallel, psychiatry has seen the emergence of the mental health exposome framework, which conceptualizes mental health as the cumulative result of multiple interacting environmental exposures across time [11]. Within this framework, light exposure stands out as both biologically potent and highly modifiable [12, 13]. Unlike many environmental risk factors, light exposure is shaped by behaviour, architecture, technology and social organization, making it a plausible target for prevention and intervention.

This review critically evaluates recent evidence linking light exposure and mental health, with an emphasis on work published over the past one to three years. We argue that light exposure should be understood as a modifiable determinant within the mental health exposome, supported by converging evidence from basic neuroscience, clinical trials, epidemiology and real-world measurement. We further highlight the bidirectional nature of the relationship between light and mental health and discuss the implications for research and clinical practice.

**Fig. 1:**
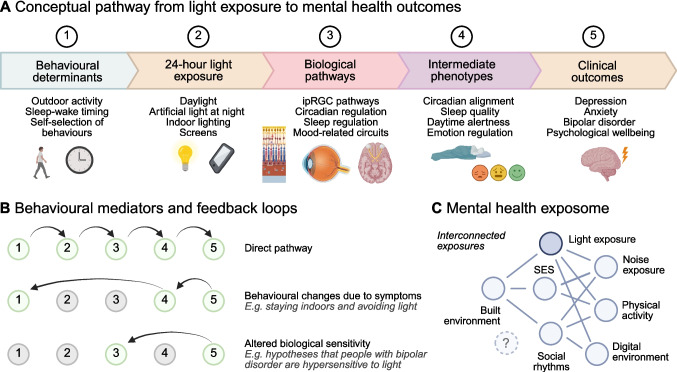
Conceptual framework linking light exposure to mental health within the mental health exposome. **A** Conceptual pathway linking behavioural determinants of light exposure to clinical mental health outcomes. Behaviour and daily routines shape 24-h light exposure (including daylight, indoor lighting and screen exposure), which in turn influences biological pathways such as intrinsically photosensitive retinal ganglion cell (ipRGC) signaling, circadian regulation and sleep processes. These biological effects contribute to intermediate phenotypes including circadian alignment, sleep quality, daytime alertness and emotion regulation, ultimately influencing clinical outcomes such as depression, anxiety and psychological wellbeing. **B** Behavioural mediators and feedback loops that modify light exposure and biological responses. For example, mental health symptoms may alter behaviour (e.g., reduced outdoor activity), which in turn changes light exposure patterns. Altered biological sensitivity to light may also contribute to feedback processes. **C** The mental health exposome, illustrating light exposure as one component of a network of interacting environmental exposures including socioeconomic context (SES), the built environment, physical activity, noise exposure, digital environments and social rhythms

To conceptualize how light exposure may influence mental health, we outline a framework linking behavioural determinants of light exposure to biological mechanisms and clinical outcomes (Fig. 1). In this framework, daily behaviour and environmental context shape patterns of 24-h light exposure, which influence circadian, sleep and neural pathways that regulate affective processes. These biological effects manifest in intermediate phenotypes such as circadian alignment, sleep quality and daytime alertness, ultimately contributing to mental health outcomes. Importantly, behavioural feedback loops and broader environmental exposures interact with light exposure within the mental health exposome.

## Evidence Linking Light Exposure and Mental Health

### Non-Visual Effects of Light

The recognition that light affects human physiology beyond image formation is now well established [3–6]. Non-visual responses to light are primarily mediated by intrinsically photosensitive retinal ganglion cells (ipRGCs), which express the photopigment melanopsin and project to a range of subcortical and cortical targets involved in circadian timing, sleep regulation and arousal [7, 14–19]. These pathways provide a biological substrate through which light can plausibly influence mental health.

Three non-visual effects are particularly relevant: circadian phase shifting, melatonin suppression and acute effects on alertness and cognition [5, 7, 20, 21]. Exposure to light in the evening and early night delays the circadian clock, whereas light exposure in the late night and early morning advances it, a relationship classically described by the human phase response curve [22–25]. In addition, light exposure in the evening and at night suppresses melatonin production, and shows similar dependence on timing, intensity, duration and spectral composition of light, with short-wavelength (“blue-enriched”) light being especially potent due to properties of the underlying melanopsin photopigment [7, 14, 26–28]. Acute alerting effects of light can occur even in the absence of circadian phase shifts, reflecting direct influences on arousal systems [8, 9, 29, 30].

From a mental health perspective, these effects matter because circadian misalignment, sleep disruption and altered arousal regulation are core features of many psychiatric conditions, including mood disorders, anxiety disorders and psychosis [31]. Importantly, the impact of light is not uniform across the day: the same light exposure may be beneficial at one circadian phase and detrimental at another [5, 22–25]. This temporal specificity complicates both research design and clinical translation, but it also offers opportunities for targeted interventions.

Recent work has further emphasized individual differences in light sensitivity, shaped by age, chronotype, prior light history and potentially genetic factors [32–36]. Such variability may help explain heterogeneous responses to light-based interventions observed in clinical studies and underscores the limitations of “one-size-fits-all” recommendations.

### Light Therapy and Daylight Exposure in Affective Disorders

The therapeutic use of light for affective disorders has a history dating back to the 1980s, when bright light therapy was first shown to alleviate symptoms of seasonal affective disorder (SAD) [37]. Since then, light therapy has been investigated across a range of mood disorders, including non-seasonal major depressive disorder, bipolar depression and perinatal depression [38, 39].

Randomized controlled trials over the past decade continue to support the efficacy of bright light therapy as both a monotherapy and an adjunct to pharmacological treatment for depressive disorders. In a randomized clinical trial in non-seasonal major depressive disorder, bright light therapy produced antidepressant effects, with the combination of light therapy combined with fluoxetine yielding the greatest symptom improvement [40]; a trial with patients with bipolar depression showed similar effects, demonstrating broader applicability [38]. Meta-analyses of randomized trials similarly report small-to-moderate reductions in depressive symptoms associated with light therapy, with effect sizes comparable to those observed for antidepressant medications in some contexts and a generally favorable side-effect profile [41]. Morning light exposure appears most effective, consistent with its circadian phase-advancing effects and the delayed circadian timing frequently observed in mood disorders.

An important evolution in the literature is the increasing recognition that “light therapy” should not be equated solely with artificial bright light boxes. Daylight and natural light exposure – through outdoor activity, architectural design and window access – are gaining attention as ecologically valid and potentially more acceptable means of delivering therapeutic light [42–44]. Recent studies have examined whether increased daytime light exposure in naturalistic settings (measured through the proxy of time spent outdoors) is associated with improved mood and reduced depressive symptoms, even outside formal treatment protocols [45].

Nevertheless, several controversies remain in the domain of light therapy. Optimal dose parameters (intensity, duration, spectral composition), treatment timing and patient selection criteria are still debated [46]. In addition, in the context of blinding participants to the treatment, the choice of appropriate placebo or control conditions in generating clinical evidence is non-trivial, as light is visible to people without visual impairment [47, 48]. Moreover, the risk of inducing mania or hypomania in individuals with bipolar disorder, while relatively low, remains a clinical concern requiring careful monitoring [49]. These unresolved issues highlight the need for better measurement of real-world light exposure and more individualized treatment approaches.

### Epidemiological Evidence from Large-Scale Cohort and Biobank Studies

Large-scale epidemiological studies have recently provided complementary evidence at the population level. Analyses of biobank and cohort datasets have reported that higher daytime light exposure is associated with lower prevalence of depressive symptoms, whereas higher nighttime light exposure is associated with increased risk of depression, anxiety and sleep disturbance [50].

These findings are notable because they capture habitual light exposure in real-world settings and across diverse populations. Some studies have incorporated objective proxies for light exposure, including wearable sensors or satellite-based measures of nighttime illumination, strengthening confidence in the associations [51]. As it is an open question to what extent the distal environmental measurements, such as from satellite measurements or stationary, ground-based measurements, can be used to estimate personal exposure, developing models to estimate exposure has been identified as a key priority [13].

From an exposome standpoint, these studies suggest that light exposure contributes to cumulative mental health risk over time. However, their observational nature limits causal inference. Reverse causation is a central concern: mental illness may alter daily behaviour, reducing daytime light exposure and increasing light exposure at night. Residual confounding by physical activity, social engagement and socioeconomic factors is also difficult to fully address.

### Neurophysiological Pathways beyond Sleep and Circadian Rhythms

Beyond circadian and sleep-related mechanisms, accumulating neurophysiological evidence suggests that light can influence mood more directly. Animal studies have demonstrated projections from ipRGCs to brain regions implicated in affective processing, including the lateral habenula, amygdala and regions of the prefrontal cortex [52, 53]. These findings challenge the notion that light affects mood solely via circadian or sleep pathways.

In humans, neuroimaging studies have shown that light exposure modulates activity in limbic and paralimbic structures involved in emotion regulation, sometimes independently of subjective alertness or sleepiness. Functional MRI studies using controlled light stimuli have reported changes in neural responses to emotional tasks under different lighting conditions [54].

Recent work has begun to link these neural effects to clinical symptoms. For example, altered light sensitivity at the neural level has been reported in individuals with mood disorders, suggesting that differences in how the brain processes light may contribute to vulnerability or resilience [55]. While this literature remains relatively small, it provides a crucial mechanistic bridge between environmental light exposure and subjective mental health outcomes.

## Using Light Exposure to Study Mental Health: Measurement and Intervention Opportunities

### Observational Characterization of Light Exposure in Mental Health Conditions

Understanding the role of light in mental health requires accurate characterization of real-world light exposure. Historically, this has been a major limitation, with most studies relying on crude proxies such as latitude, season or self-reported time outdoors. The increasing availability of wearable light loggers represents a major methodological advance in this area.

**Fig. 2:**
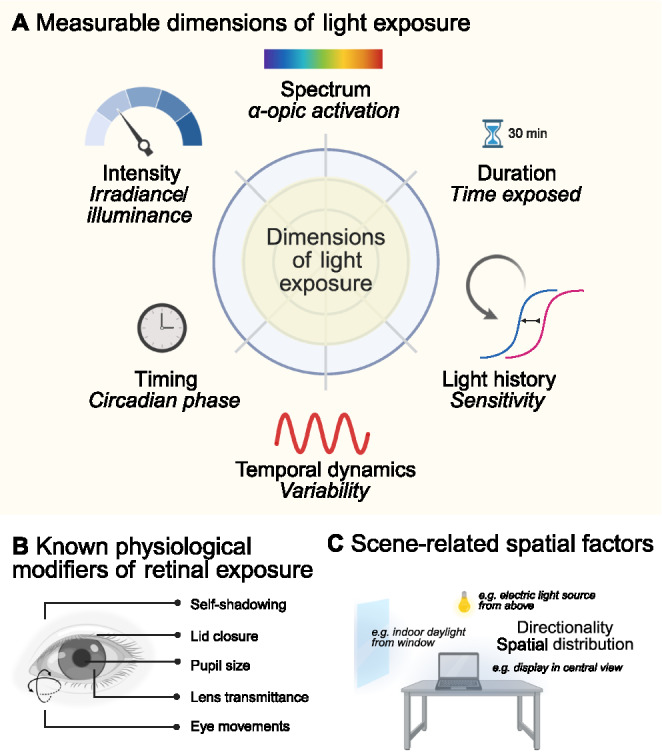
Measurement and determinants of real-world light exposure. **A** Measurable dimensions of light exposure that can be captured using wearable light loggers, including intensity (irradiance or illuminance), spectral composition (e.g., α-opic activation), exposure duration, timing relative to circadian phase, temporal variability, and prior light history influencing sensitivity. **B** Physiological modifiers that influence the amount of light reaching the retina, including pupil size, eyelid position, lens transmission, eye movements and self-shadowing effects. **C** Scene-related spatial factors that influence retinal light exposure, such as the spatial distribution and directionality of light sources within the visual environment (e.g., daylight from windows, overhead electric lighting, or displays within the field of view)

Light exposure is a multidimensional environmental signal that can be characterized across several measurable dimensions (Fig. 2). These include the intensity, spectral composition, duration and timing of exposure, as well as temporal dynamics and prior light history. In addition, the amount of light reaching the retina depends on physiological factors such as pupil size and eye movements, as well as spatial characteristics of the visual environment including the directionality and distribution of light sources [56].

**Fig. 3:**
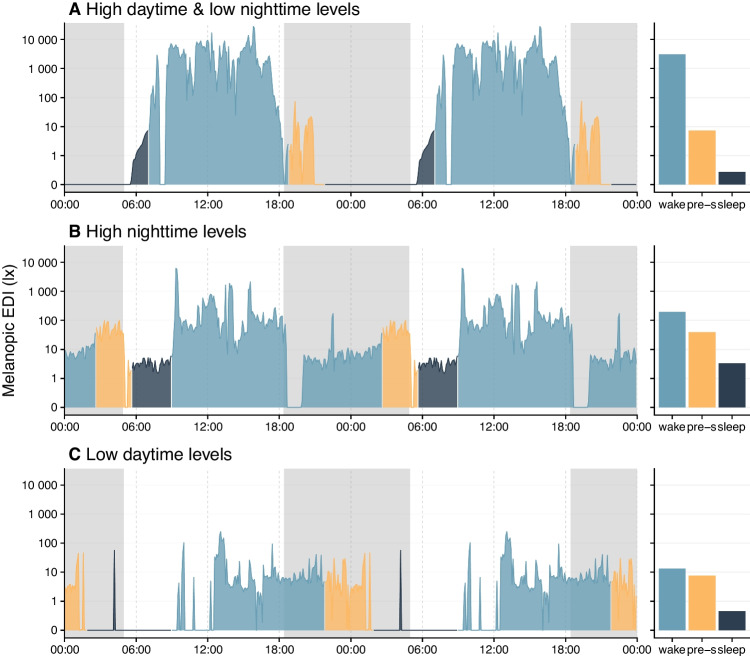
Exemplary real-world light exposure patterns illustrating different daily light exposure phenotypes. Continuous measurements of melanopic equivalent daylight illuminance (EDI) across two consecutive days are shown for three representative exposure patterns. **A** High daytime light exposure combined with low nighttime light exposure, representing a pattern generally considered favorable for circadian alignment. **B** Elevated nighttime light exposure alongside moderate daytime light exposure. **C** Low daytime light exposure, reflecting limited exposure to bright environmental light during the day. Bar plots on the right summarize average melanopic EDI during the wake period, the three hours preceding sleep, and the sleep period. Data were collected in San José, Costa Rica [66], within the MeLiDos Project [67] deploying a standard multi-day light exposure measurement protocol [68]. Panel A: 49-year-old female participant, data from 12 July 2025 (S010 in dataset); panel B: 23-year-old male participant, data from 19 June 2025 (S003); panel C: 29-year-old male participant, data from 5 July 2025 (S009)

Wearable devices can capture continuous light exposure data across days or weeks, enabling detailed analyses of intensity, timing and variability of light exposure in daily life [57–63]. Wearable light loggers make it possible to capture several of these exposure dimensions simultaneously in real-world settings (Fig. 2A). Figure 3 shows example data with different qualitative daytime, evening and nighttime light exposure patterns. Recent observational studies have used such devices to compare light exposure patterns across populations, revealing substantial differences [64, 65].

In psychiatric research, wearable light loggers offer the opportunity to characterize how light exposure differs in individuals with depression, bipolar disorder or anxiety compared to healthy controls [69, 70], and the evidence base suggests that affected individuals experience lower light levels than healthy controls. This difference may indicate the need to increase light exposure through behavioural or technological means. Fundamentally, such data can help disentangle whether altered light exposure is a feature of the disorder, a risk factor or both; and additionally can also indicate the directionality of interventions. Importantly, these approaches align with a broader move toward ecological momentary assessment and real-world phenotyping in mental health research.

## Monitoring Compliance and Dose in Light Therapy Studies

**Fig. 4:**
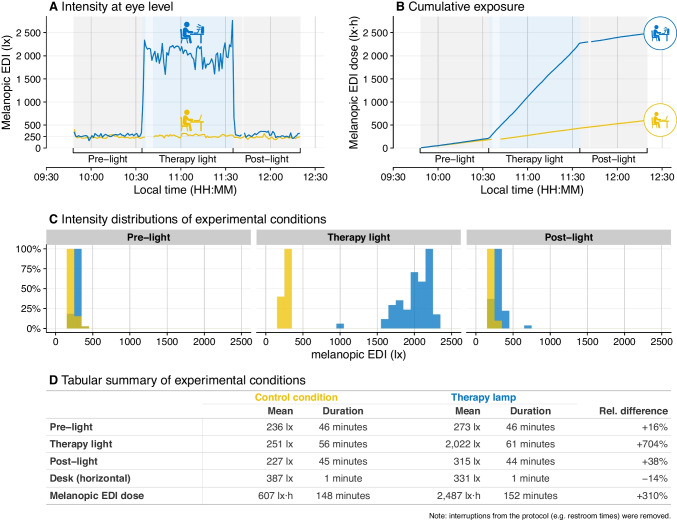
Objective measurement of light exposure during a light therapy intervention compared to an inactive control condition. **A** Time series of melanopic equivalent daylight illuminance (EDI) measured at eye level during a morning experimental session, showing the pre-exposure, therapy light, and post-exposure phases for a light therapy lamp condition compared with a control condition in the same environment. **B** Cumulative melanopic EDI dose across the session, illustrating the substantially greater light dose delivered during the therapy condition. **C** Distributions of melanopic EDI values across the three experimental phases (pre-light, therapy light, post-light) for the control and therapy conditions. **D** Summary table reporting mean melanopic EDI, exposure duration, and total melanopic dose across conditions

A second, and arguably underappreciated, application of wearable light loggers is in light therapy trials. Compliance with prescribed light therapy regimens is often assumed rather than measured and actual “dose received” can differ substantially from “dose prescribed” [71] (Fig. 4).

By objectively recording light exposure, researchers can verify whether participants are receiving the intended intensity and timing of light, identify deviations from protocol and relate these deviations to treatment outcomes [71]. This approach mirrors developments in pharmacology, where therapeutic drug monitoring has long been recognized as essential for interpreting treatment effects.

Studies have begun to incorporate wearable light measurement into light therapy trials, revealing wide variability in exposure [72–74]. Such findings may help explain inconsistent results across trials and highlight the need for more rigorous exposure assessment.

### Toward Closed-Loop and Personalized Light Interventions

Looking ahead, one of the most promising developments is the prospect of closed-loop light intervention systems (e.g., [75]) deploying just-in-time interventions. These systems could combine continuous measurement of light exposure, sleep, activity or circadian phase with algorithms that generate personalized recommendations for optimal light exposure [76, 77].

In principle, such systems could adapt light exposure advice based on an individual’s chronotype, daily schedule and mental health status, updating recommendations in real time. Early proof-of-concept studies have demonstrated the feasibility of such approaches [78], though robust clinical evidence is still emerging.

From a mental health perspective, closed-loop systems offer a way to move beyond static prescriptions toward dynamic, context-sensitive interventions. However, they also raise important questions about evidence thresholds, user adherence and the risk of overmedicalizing everyday light exposure.

## A Converging Evidence Framework and Conceptual Challenges Integrating Evidence Across Levels of Analysis

Taken together, the evidence reviewed here points toward a converging picture: light exposure influences mental health through multiple, interacting pathways and this influence is detectable across experimental, epidemiological and mechanistic domains. No single line of evidence is decisive on its own, but their convergence strengthens the overall inference.

Randomized trials demonstrate that manipulating light can improve mood in clinical populations. Epidemiological studies show that habitual light exposure patterns are associated with mental health outcomes at the population level. Neurophysiological studies provide plausible mechanisms linking light to affective processing. Measurement advances enable increasingly precise characterization of exposure. Evidence from randomized trials and meta-analyses indicates that increasing morning bright light can reduce depressive symptoms, while large cohort studies suggest that greater habitual daytime or outdoor light exposure is associated with lower depressive symptom burden and lower incident depression risk. Together, these strands form a coherent, though still incomplete, evidence base.

Light exposure therefore is a core component of the *mental health exposome*, the totality of environmental exposures that shape mental health across the lifespan. Within this framework, light represents a biologically potent, temporally structured and behaviourally mediated exposure that interacts with sleep, circadian rhythms, daily routines and social context.

Key open questions and methodological challenges and emerging ways forward are summarized in Text Box 1.

**Text Box 1:** Open questions, challenges and ways forward

Despite growing evidence, integrating light exposure into mental health research and practice faces several unresolved challenges that require deliberate methodological and conceptual advances.
**1.** **Causality in a bidirectional system**. Light exposure both shapes and is shaped by mental health–related behaviour. Treating light as a unidirectional risk factor is therefore misleading. *Way forward:* Prioritize longitudinal designs, within-person analyses, and intervention studies with objective exposure measurement to explicitly model feedback loops rather than attempting to “control them away.”
**2.** **Bridging short-term responses and long-term outcomes**. Most mechanistic evidence concerns acute or short-term effects of light (e.g., alertness, melatonin suppression, neural responses), whereas mental health outcomes unfold over weeks to years. How transient light responses accumulate into long-term risk or resilience remains largely unknown. *Way forward:* Link high-resolution, short-term light exposure and response measures to longer-term trajectories using repeated measures, cumulative exposure metrics and mechanistically informed models.
**3.** **What constitutes a meaningful light “dose”?** There is no single biologically relevant dose of light: timing, intensity, duration, spectrum and prior exposure interact nonlinearly. This complicates both epidemiology and intervention design. *Way forward:* Move from single-metric exposure proxies toward multidimensional, data-driven light metrics tailored to specific outcomes.
**4.** **Individual differences as signal, not noise.** Large interindividual variability in light sensitivity undermines one-size-fits-all recommendations but also offers explanatory power. *Way forward:* Treat variability as a primary outcome by stratifying analyses by chronotype, age and baseline light exposure, and by developing personalized intervention frameworks.
**5.** **Light rarely acts alone.** Light exposure is tightly coupled to physical activity, social rhythms, sleep timing and the built environment, raising persistent confounding concerns. As part of the mental health exposome, relevant co-exposures need to be considered. *Way forward:* Embed light within exposome-aware models that jointly consider co-exposures instead of isolating light post-hoc.

## Clinical Implications

From a clinical perspective, the accumulating evidence suggests several practical implications. First, light exposure should be considered a relevant contextual factor in psychiatric assessment, alongside sleep, activity and social rhythms. Simple questions about daytime outdoor exposure and nighttime light habits may yield clinically meaningful information.

Second, light-based interventions – whether formal light therapy or structured advice on daylight exposure and evening light reduction – represent low-risk, potentially high-yield adjuncts to standard treatments. While not a panacea, they may enhance treatment response, particularly in mood disorders.

Third, clinicians should be aware of individual differences in light sensitivity and circadian timing. What is beneficial for one patient may be ineffective or counterproductive for another (because of, e.g., visual discomfort). Personalized approaches, informed by emerging measurement tools, are likely to be more effective than uniform recommendations.

Finally, the bidirectional nature of the relationship underscores the importance of timing. Interventions aimed at improving light exposure may be most effective when integrated with broader efforts to stabilize daily routines and sleep–wake patterns.

## Conclusion

The relationship between light exposure and mental health is supported by a growing and increasingly diverse body of evidence. Over the past several years, advances in epidemiology, neuroscience and measurement technology have strengthened the case for light as a meaningful environmental factor in psychiatric health.

At the same time, important challenges remain. Causality is difficult to establish, individual differences are substantial, and the relationship is likely fundamentally bidirectional. Addressing these challenges will require integrative, longitudinal and personalized approaches.

Nevertheless, light exposure stands out as a modifiable factor with clear biological relevance and clinical potential. As research continues to converge across disciplines, light is set up to move from the periphery toward the mainstream of mental health science and practice.

## Key References

- Burns, A.C., et al., Day and night light exposure are associated with psychiatric disorders: an objective light study in > 85,000 people. *Nature Mental Health*, 2023. **1**(11): p. 853–862.
  - This large-scale population study uses objectively measured light exposure to demonstrate opposite associations of daytime and nighttime light with multiple psychiatric outcomes. It provides some of the strongest epidemiological evidence to date linking habitual light exposure patterns to mental health.
- Lin, J., et al., *Association of time spent in outdoor light and genetic risk with the incidence of depression.* Transl Psychiatry, 2023. **13**(1): p. 40.
  - This prospective study shows that greater outdoor light exposure is associated with a reduced risk of depression, even after accounting for genetic vulnerability. It strengthens the case for daylight exposure as a potentially protective, modifiable environmental factor.
- Hartmeyer, S.L. and M. Andersen, *Towards a framework for light-dosimetry studies: Quantification metrics.* Lighting Research & Technology, 2023. **56**(4): p. 337–365.
  - This paper provides a rigorous framework for quantifying light exposure using multidimensional metrics that account for timing, intensity, duration, and spectral composition. It is a key methodological reference for moving beyond single-metric proxies toward biologically meaningful light “dose” definitions in mental health and exposome research.

## Data Availability

All code and data are available at https://github.com/tscnlab/SpitschanEtAl_CurrPsychiatryRep_2025.
